# Thromboembolic complications in critically ill COVID-19 patients are associated with impaired fibrinolysis

**DOI:** 10.1186/s13054-020-03401-8

**Published:** 2020-12-07

**Authors:** Jan Matthias Kruse, Abakar Magomedov, Annika Kurreck, Frédéric H. Münch, Roland Koerner, Julian Kamhieh-Milz, Andreas Kahl, Inka Gotthardt, Sophie K. Piper, Kai-Uwe Eckardt, Thomas Dörner, Daniel Zickler

**Affiliations:** 1 Department of Nephrology and Medical Intensive Care, Charité – Universitätsmedizin Berlin, Augustenburger Platz 1, 13353 Berlin, Germany; 2 Department of Hepatology and Gastroenterology, Charité – Universitätsmedizin Berlin, Berlin, Germany; 3 Department of Hematology and Oncology, Charité – Universitätsmedizin Berlin, Berlin, Germany; 4 Institute for Transfusion Medicine, Charité – Universitätsmedizin Berlin, Berlin, Germany; 5 Wimedko GmbH, Berlin, Germany; 6 Institute of Biometry and Clinical Epidemiology, Charité – Universitätmedizin Berlin, Berlin, Germany; 7 Berlin Institute of Health (BIH), Berlin, Germany; 8 Department of Rheumatology Und Clinical Immunology, Charité – Universitätsmedizin Berlin, Berlin, Germany; 9 Deutsches Rheumaforschungszentrum (DRFZ), Berlin, Germany

**Keywords:** COVID-19, Coagulopathy, Hypofibrinolysis, ROTEM, D-dimers

## Abstract

**Background:**

There is emerging evidence for enhanced blood coagulation in coronavirus 2019 (COVID-19) patients, with thromboembolic complications contributing to morbidity and mortality. The mechanisms underlying this prothrombotic state remain enigmatic. Further data to guide anticoagulation strategies are urgently required.

**Methods:**

We used viscoelastic rotational thromboelastometry (ROTEM) in a single-center cohort of 40 critically ill COVID-19 patients.

**Results:**

Clear signs of a hypercoagulable state due to severe hypofibrinolysis were found. Maximum lysis, especially following stimulation of the extrinsic coagulation system, was inversely associated with an enhanced risk of thromboembolic complications. Combining values for maximum lysis with D-dimer concentrations revealed high sensitivity and specificity of thromboembolic risk prediction.

**Conclusions:**

The study identifies a reduction in fibrinolysis as an important mechanism in COVID-19-associated coagulopathy. The combination of ROTEM and D-dimer concentrations may prove valuable in identifying patients requiring higher intensity anticoagulation.
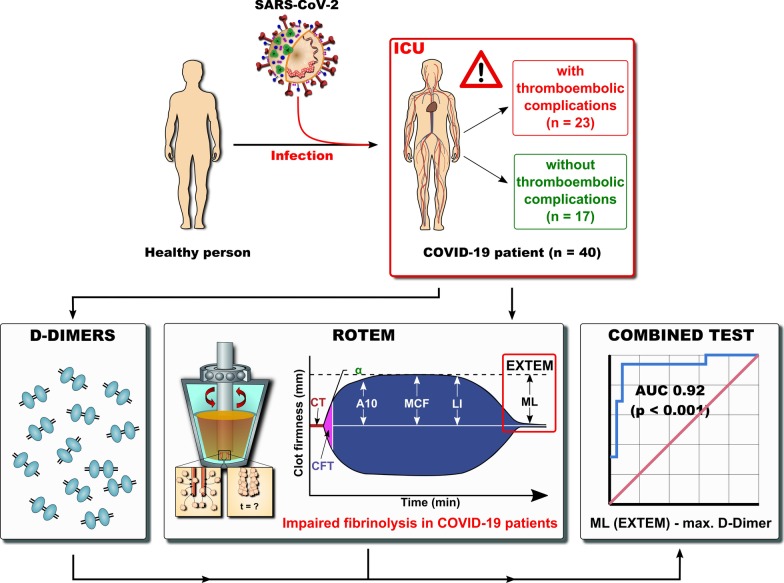

## Background

The novel severe acute respiratory syndrome coronavirus 2 (SARS-CoV-2) causing coronavirus disease 2019 (COVID-19) has led to a global pandemic posing a major threat to humans [1]. More than 500 000 deaths related to COVID-19 have been so far reported [2].

SARS-CoV-2 primarily affects the respiratory system with a widely heterogeneous clinical presentation, ranging from none or minimal symptoms to significant hypoxia with viral pneumonia, potentially leading to severe acute respiratory distress syndrome (ARDS) and cytokine storm [3]. ARDS with related lung injury is considered one of the main causes of death in COVID-19 patients [4].

However, there is emerging evidence that involvement of other pathomechanisms contributes to morbidity and mortality. Both clinical and autopsy studies have revealed a high incidence of venous and arterial thromboembolic events, including pulmonary embolism, even in patients receiving therapeutic anticoagulation [5–7]. These findings have led to recommendations for higher anticoagulation targets; however, it remains unclear which patients are at increased risk and require anticoagulation [8]. While fibrinogen and D-dimer levels are frequently elevated, neither parameter reliably identifies patients at an increased risk of thromboembolic complications [8]. Although different markers of hypercoagulation have been reported among COVID-19 patients [6, 9], the exact mechanisms underlying the prothrombotic state in these patients remain unclear so far [10, 11]. In particular, it has not been clarified to which extent increased procoagulation and/or impaired fibrinolysis is involved.

In addition to conventional laboratory parameters, rotational thromboelastometry (ROTEM) provides evidence for net coagulation capacity and insight into clot formation time, clot firmness and fibrinolysis in the critically ill patients [12]. Here we report ROTEM data in 40 consecutive, severely ill COVID-19 patients treated in two tertiary intensive care units (ICUs) and assessed the association with thromboembolic complications.

## Methods

### Coagulation tests

After admission to our ICUs, blood samples were drawn and viscoelastic tests were performed once with citrated blood using a ROTEM sigma point-of-care device (Tem International, Munich, Germany) [13]. In each patient, intrinsically (contact activation, INTEM) and extrinsically (tissue factor activation, EXTEM) activated test assays were performed to analyze the clot dynamics in both coagulation pathways. Furthermore, FIBTEM and HEPTEM were performed. In the FIBTEM, platelets are inactivated with cytochalasin D to enable isolated evaluation of fibrinogen in clot firmness. The heparin effect was determined by comparing the clotting time of the INTEM with the clotting time of the HEPTEM, where heparinase is added.

**Fig. 1:**
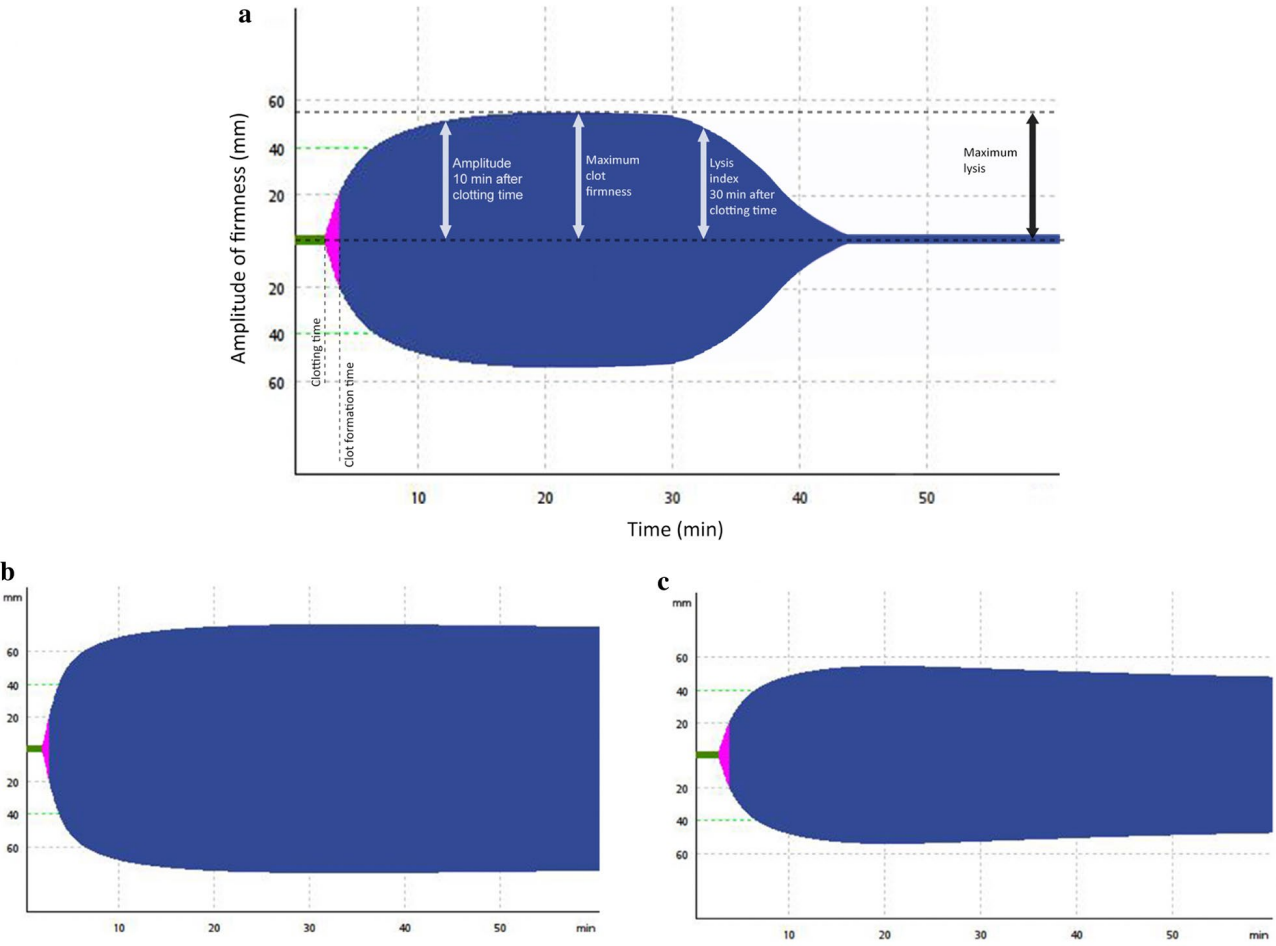
**a** All measured values in ROTEM analysis, including clotting time (CT [s]), clot formation time (CFT [s]), maximum clot firmness (MCF [mm]) and maximum lysis (ML [%(range)]). **b** A reduction of fibrinolysis in a COVID-19 patient with a thromboembolic event; the clot amplitude remains unchanged until the end. **c** A physiological fibrinolysis pattern in a healthy person, reflected by the subtle decrease of the MCF during the measurement

The following ROTEM variables were analyzed: clotting time defined as the time until initiation of clotting; clot formation time (seconds until a clot strength reaches 20 mm), reflecting the kinetics of clot formation; maximum clot firmness (MCF) defined as the maximum amplitude of clot firmness; maximum lysis (ML; %) defined as the difference between MCF and the lowest clot amplitude after MCF, reflecting fibrinolytic activity (Fig. 1).

Additional routine laboratory tests performed according to standardized protocols comprised hemoglobin concentration, white blood cell count, platelet count, prothrombin time (PT), international normalized ratio (INR), activated partial thromboplastin time (aPTT) and inflammatory parameters (see Table 2). The levels of tissue-type plasminogen activator (t-PA), plasminogen activator inhibitor-1 (PAI-1) and plasminogen were determined using commercial ELISA Kits (t-PA Antigen ELISA Kit, PAI-1 Antigen ELISA Kit, Glu-Plasminogen, TECHNOZYM®/Technoclone).

To combine the parameters maximum D-dimers (mg/l) and ML (%), the difference (maximum D-dimers—ML EXTEM) was calculated and analyzed.

### Anticoagulation therapy

In our Intensive care units, all patients included in this trial were treated with either low molecular weight heparin or in the case of ECMO therapy with argatroban. We aimed for a PTT of 50–55 s (normal 26–40 s), and in patients with thromboembolic events we aimed for a PTT of 60–80 s.

### Ultrasound

We performed ultrasound examinations in all patients (GE Vivid S70 ultrasound machine with a 9L-D probe) to screen for venous thrombosis, focusing on the jugular, subclavian, brachial, femoral and popliteal veins upon admission to our ICU and subsequently at least once weekly.

### Ethics

The study was approved by the ethics committees of Charité – Universitätsmedizin Berlin (EA4/115/20).

### Statistics

Statistical analyses were performed using IBM^®^ SPSS^®^ Statistics version 26 (New York, USA). The descriptives are provided as median with limits of the interquartile range (IQR) for continuous variables or as absolute and relative frequencies for categorical variables.

Continuous data were primarily right skewed. Therefore, the Mann–Whitney *U* test was used to compare differences between patient groups in continuous variables, while Chi-square test was used for categorical data. A two-sided significance level of 0.05 was applied without adjustment for multiple comparison. All *p* values constitute exploratory data analyses and do not allow for confirmatory generalization of results. To evaluate the strength of different ROTEM variables to distinguish between patients with and without thromboembolic events, receiver operating characteristic (ROC) analysis was carried out including area under the curve measures (AUC) with 95% confidence intervals (CI). Sensitivity, specificity and accuracy (percentage of correctly classified patients) are reported.

## Results

### Characteristics of the cohort

Forty consecutive patients with COVID-19 confirmed by polymerase chain reaction in throat swabs were admitted to two ICUs within our department between March 25th and May 11th. All patients received viscoelastic testing using the ROTEM system and were included in the analysis, which was censored on May 11th.

Table 1 shows baseline characteristics of the study cohort. As most patients were referred from community hospitals within a regional network, patients were mostly severely ill with a median sequential organ failure assessment (SOFA) score of 9 and a mean acute physiology and chronic health evaluation (APACHE) II of 28 points. Mechanical ventilation via either endotracheal tube or tracheostomy was administered to 78% of patients, whereas extracorporeal membrane oxygenation was required for 25% and kidney replacement therapy for 53% of patients. Evidence for macrothromboembolic events was found in 23 of 40 patients (58%). In five patients, we identified thromboembolic events upon admission to our ICUs (*N* = 3 prediagnosed pulmonary emboli, *N* = 2 deep venous thrombosis). Nineteen patients developed thromboembolic complications during the ICU stay, comprising deep vein thrombosis (*N* = 14), pulmonary embolism (*N* = 4), ischemic stroke (*N* = 3), complete thrombosis of the ECMO-circuit requiring emergency circuit-change (*N* = 1) and a clotted ECMO cannula (*N* = 1).

**Table 1:** Patient characteristics of total cohort and subcohorts with and without thromboembolic events

	Cohort (*n* = 40)		Thromboembolic events (*N* = 23)		No thromboembolic events (*N* = 17)		*p* value
Age (years, (median, [IQR]))	67	[57.3–76.6]	66	[56–76]	68	[62–77.5]	ns
Gender, male (*n*, %)	35	87.5%	20	87%	15	88%	ns
BMI, kg/m^2^ (median, [IQR])	28.1	[24.8–32.8]	27.8	[24.2–33]	28.7	[25.7–32.3]	ns
Duration of ICU stay, days (median, [IQR])	39.5	[24–54.25]	42	[28–58]	25	[8.5–47.5]	0.05
Death during ICU stay (*n*, %)	11	27.5%	9	39.1%	2	11.8%	0.58
Intubation (*n*, %)	31	77.5%	20	87%	11	65%	ns
ECMO (*n*, %)	10	25%	9	39.1%	1	6%	ns
CRRT (*n*, %)	21	52.5%	16	69.6%	5	29.4%	0.013
SOFA score (median, [IQR])	9	[6.3–11.8]	10	[6–11]	8	[4.5–11]	ns
SIC score (median, [IQR])	3	[2–4]	3	[2–4]	3	[2–4]	ns
APACHE score (median, [IQR])	28	[22–33]	29	[23–34]	26	[19–31.8]	ns
Preexisting conditions							
Coronary artery disease (*n*, %)	9	22.5%	6	26%	3	18%	ns
Hypertension (*n*, %)	25	62.5%	14	61%	11	65%	ns
Diabetes mellitus/insulin resistance (*n*, %)	13	32.5%	10	43%	3	18%	ns
Chronic kidney disease (*n*, %)	7	17.5%	6	26%	1	6%	ns
Chronic dialysis (*n*, %)	1	2.5%	1	4%	0	0%	ns
Lung disease (*n*, %)	7	17.5%	6	26%	1	6%	ns

*ECMO* Extracorporeal membrane oxygenation, *SOFA* sequential organ failure assessment, *CRRT* continuous renal replacement therapy, *SIC* sepsis-induced coagulopathy, *APACHE* acute physiology and chronic health evaluation

### Laboratory parameters

Table 2 shows laboratory parameters for the study cohort and in patients with and without thromboembolic events. Hematological parameters were similar in both patient groups. Patients with thromboembolic events had a significantly higher maximum C-reactive protein (CRP) value, with a median value of 341 mg/l [IQR 261.1–370.7] versus 261.1 mg/l [IQR 175.3–312.9], respectively (*p* = 0.002). Other markers of inflammation such as procalcitonin (PCT), ferritin and interleukin-6 did not differ significantly between groups.

**Table 2:** Laboratory parameters of total cohort and subcohorts with and without thromboembolic events

	Cohort (*N* = 40)		Thromboembolic event				
			Yes (*N* = 23)		No (*N* = 17)		
	Median	[IQR]	Median	[IQR]	Median	[IQR]	*p* value
Laboratory variables (normal values)							
Haemoglobin (12·5–17·2 g/dL)	10.1	[8.5–11.2]	9.70	[8.3–10.8]	10.4	[9.3–11.9]	ns
White blood cells (3·5–10·5/nl)	10.13	[7.5–13.7]	10.63	[7.4–16]	9.58	[6.6–12.1]	ns
Platelet count (150–370/nl)	193.5	[131.3–316.3]	181	[116–306]	209	[178–325.5]	ns
Prothrombin time (70–130%)	74.5	[62.8–86]	79	[61–83]	71	[63.5–87.5]	ns
INR (0·9–1·25)	1.2	[1.1–1.4]	1.18	[1.1–1.4]	1.26	[1.1–1.4]	ns
PTT (26–40 s)	45.65	[39.4–56.1]	51.10	[40.8–57.4]	41.1	[38.7–54.2]	ns
Fibrinogen (1·6–4 g/l)	6.67	[4.7–7.7]	6.72	[5.0–7.8]	6.1	[4.6–7.9]	ns
D-dimers (< 0·5 mg/l)	3.95	[2.6–5.9]	4.84	[3.5–7.2]	3.06	[2.3–3.9]	0.003
max. D-dimers (< 0·5 mg/l)	8.25	[3.6–16.2]	11.57	[8.2–18.4]	3.98	[2.6–6.4]	< 0.001
Procalcitonin (0·5 µg/l)	0.57	[0.2–2.5]	0.81	[0.4–4.7]	0.24	[0.2–1.3]	ns
CRP (< 0·5 mg/l)	123.8	[84.3–216.5]	130	[86–273.7]	111	[79.3–185]	ns
max. CRP (< 0·5 mg/l)	312.9	[208.3–343.9]	341.4	[261.1–370.7]	261.05	[175.3–312.9]	0.002
IL-6 (< 7 ng/l)	103	[35·6–230]	88	[27.7–340]	153	[53.7–206.5]	ns
max. IL-6 (< 7 ng/l)	558.6	[178.8–1792.3]	550	[174–2475]	567.2	[186.5–1196.5]	ns
Ferritin (30–400 µg/l)	1636	[1067.8–4028.5]	1663	[1218.5–4655]	1567	[720–3662]	ns
max. Ferritin (30–400 µg/l)	2523.2	[1536.7–6635.1]	2781.5	[1854.7–7996.2]	2028.4	[922.9–4893.4]	ns
tPA (2–8 µg/l)	1	[0.9–5.5]	1	[0.9–3.6]	2	[0.9–9.9]	ns
PAI-1 (7–43 ng/ml)	36	[17–70]	31	[12–61]	42.50	[25.3–87]	ns
tPA/PAI-1	0.053	[0.02–0.18]	0.05	[0.02–0.14]	11	[0.03–0.24]	ns
Antithrombin III (80–120%)	79	[58.5–96.5]	75.5	[56.8–84]	94	[66.5–110]	ns
Factor VIII (50–150%)	258	[190.5–319.5]	260	[219.5–355]	222	[149.5–289.5]	ns
Plasminogen (80–120%)	88	[72.8–114]	82	[72.8–109.8]	101	[70.8–129.8]	ns
ROTEM variables							
FIBTEM CT (s)	88.5	[78–97.8]	89	[78–102]	88	[75.5–96]	ns
FIBTEM CFT (s)	68	[51–104]	64.5	[54–95.8]	71	[47–165]	ns
FIBTEM MCF (mm)	34.5	[27.3–39.5]	35	[27–38]	34	[27–40]	ns
EXTEM CT (s)	86	[69.5–99.8]	84	[69–96]	86	[70.5–107.5]	ns
EXTEM CFT (s)	46.5	[40–60.5]	47	[40–61]	45	[40.5–56.5]	ns
EXTEM MCF (mm)	75	[70.3–78]	75	[69–78]	76	[72.5–78.5]	ns
INTEM CT (s)	208	[181.3–227.5]	215	[197–251]	189	[171.5–212]	0.005
INTEM CFT (s)	50.5	[39.5–61.8]	56	[39–63]	45	[39.5–60.5]	ns
INTEM MCF (mm)	74	[69–77]	74	[65–77]	73	[69.5–78]	ns
HEPTEM CT (s)	188.5	[170.5–208.3]	193	[173–209]	173	[159–206]	ns
HEPTEM CFT (s)	41	[35.5–56.5]	40	[34–60]	42	[37–51]	ns
HEPTEM MCF (mm)	73	[67.5–75.3]	73	[66–76]	71	[71–75]	ns
ML, EXTEM (%)	3	[1.3–5.8]	3	[0–5]	5	[3.5–8]	0.001
ML, INTEM (%)	3	[1–6]	2	[0–3]	6	[2.5–6]	0.001

Unless values are designated as maximum values during the ICU stay, these parameters were determined on the day, when ROTEM analysis was performed, after admission to our ICUs

*CT* clotting time, *CFT* clot formation time, *MCF* maximum clot firmness, *ML* maximum lysis

Analyses of the coagulation parameters revealed no significant differences between the groups with the exception of a prolonged PTT in the group with thromboembolic events. Patients had significantly elevated levels of fibrinogen without significant differences between groups.

Moreover, the median initial D-dimer levels were 4.84 mg/l [IQR 3.5–7.2] in the group with thromboembolic complications in comparison with 3.06 mg/l [IQR 2.3–3.9] in the group without thromboembolic complications (*p* = 0.003).

### ROTEM parameters

Substantial abnormalities in the ROTEM analysis were found in the overall cohort. Maximum clot firmness in INTEM, EXTEM, FIBTEM and HEPTEM was markedly elevated in the entire cohort compared to reference values with median values of 74 mm [IQR 69–77], 75 mm [IQR 70.3–78], 34.5 mm [IQR 27.3–39.5] and 73 mm [IQR 67.5–75.3], respectively. Of note, there was no significant difference in these parameters between the subgroups with and without thromboembolic complications. However, the median clotting time detected in INTEM was significantly longer in the group of patients with thromboembolic complications: 215 s [IQR 197–251] versus 189 s [IQR 171.5–212]; *p* = 0.005. Clotting times in FIBTEM, EXTEM and HEPTEM showed no significant differences between groups.

**Fig. 2:**
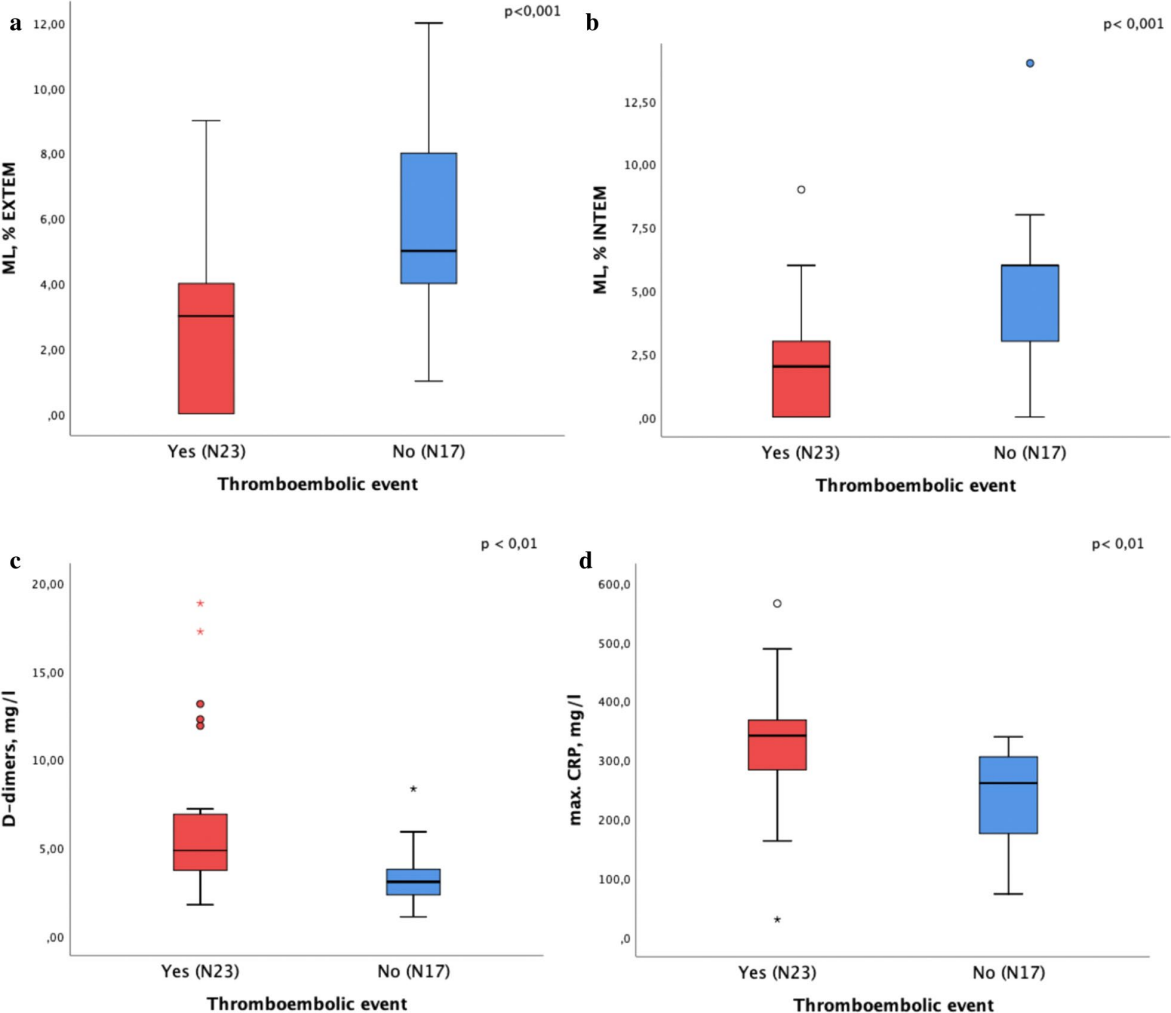
**a** Maximum lysis (ML) in EXTEM, **b** maximum lysis (ML) in INTEM, **c** D-dimers on the day of ROTEM and **d** max. CRP in COVID-19 patients with and without thromboembolic complications

Figure 2 depicts ML in INTEM and EXTEM. Under both conditions, ML was reduced and significantly lower in the group with thromboembolic complications (INTEM median 2% [IQR 0–3.0] versus 6% [IQR 2.5–6]; *p* = 0.001; EXTEM median 3% [IQR 0–5] versus 5% [IQR 3.5–8], *p* = 0.001), indicating substantially impaired fibrinolysis in both groups. This was observed to be more pronounced in patients with thromboembolic complications.

### ROC analysis to distinguish patients with and without thromboembolic complications

**Fig. 3:**
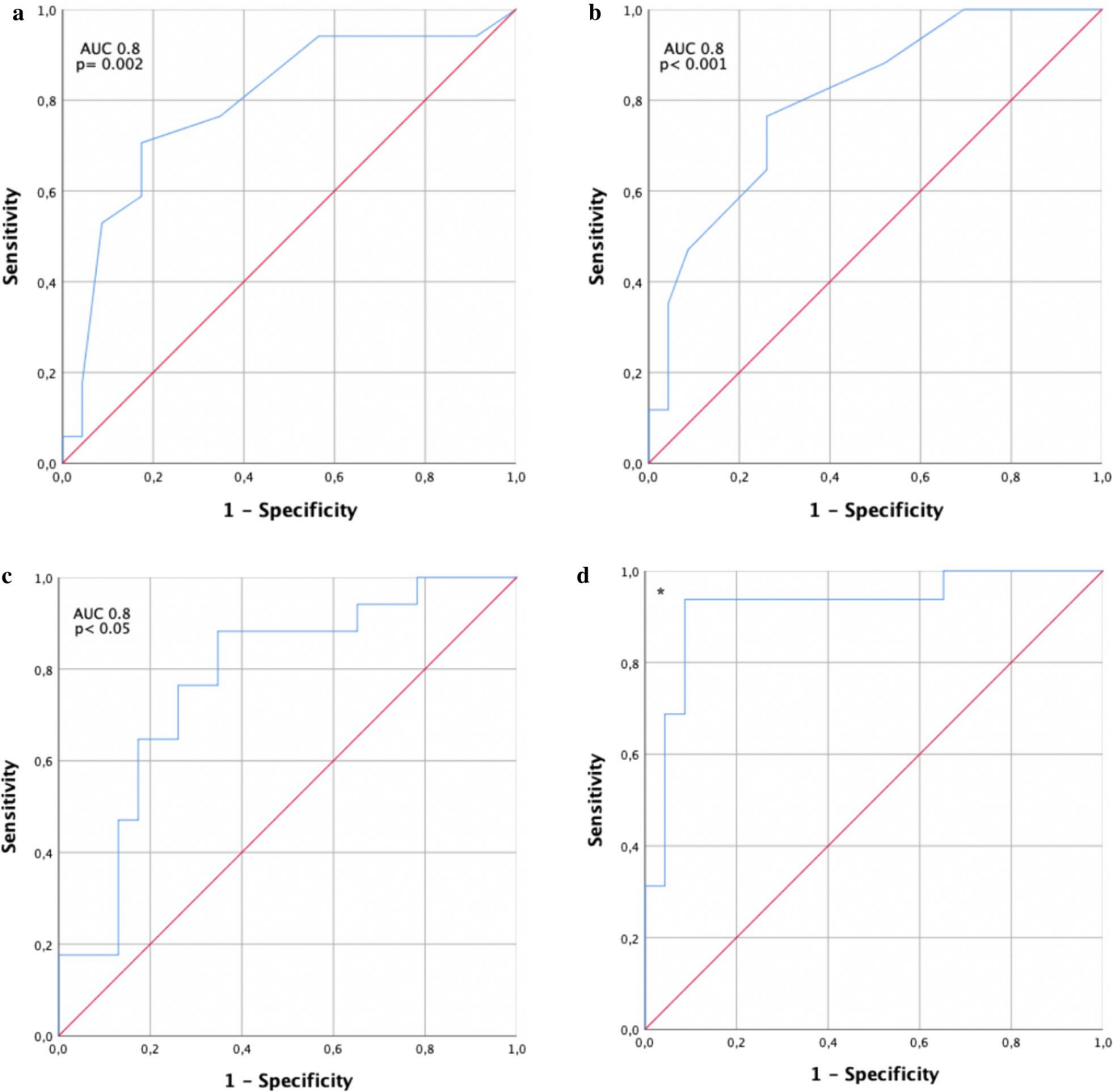
ROC analysis of **a** maximum lysis (ML) in EXTEM, **b** D-dimer and **c** ML INTEM **d** difference of ML in EXTEM and max. D-dimer for prediction of thromboembolic events in our cohort [*AUC of 0·92 (*p* < 0.001)]

Based on the above findings, we evaluated the potential of different ROTEM variables to distinguish between patients with and without thromboembolic events using ROC analysis (Fig. 3). Maximum lysis in EXTEM resulted in an area under the curve (AUC) of 0.8 [95% CI 0.7–0.9] for thromboembolic events (*p* = 0.001), while the ML in INTEM resulted in an AUC of 0.79 [95% CI 0.6–0.9] (*p* = 0.002). D-dimers showed an AUC of 0.78 [95% CI 0.6–0.9], and maximum D-dimers had an AUC of 0.82 [95% CI 0.7–1.0]. Combined analysis showed that the difference in D-dimers and ML EXTEM resulted in an AUC of 0.92 [95% CI 0.8–1].

## Discussion

This study provides evidence that hypofibrinolysis is an important contributor to the hypercoagulable state in COVID-19 patients. Maximum lysis assessed in ROTEM analysis, especially in the EXTEM analysis, was reduced more profoundly in patients with thromboembolic events. Based on these observations, we propose that ROTEM analysis is useful for patient stratification according to their prothrombotic risk. In particular, combined consideration of ROTEM maximum lysis and D-dimers may identify patients that benefit from therapeutic anticoagulation.

In this small cohort of severely ill COVID-19 patients, we observed thromboembolic complications in more than 50% of patients. Analysis of routine coagulation parameters should be interpreted with caution, as many of the patients were treated with therapeutic anticoagulation. However, in accordance with previous studies, fibrinogen and factor VIII were elevated in our cohort and D-dimers were significantly elevated in the subgroup with thromboembolic complications [14]. Other conventional markers of the coagulation system showed no significant differences between the two groups.

In contrast to individual parameters, viscoelastic methods, such as thromboelastography and ROTEM, permit functional evaluations by recording most components of the coagulation process in vitro in the presence of cellular blood components. This provides insight into the different coagulation phases, including the initiation, formation and stabilization of a clot, and finally, clot lysis. The influence of the endothelium as an important co-factor of coagulation, however, is not directly reflected in ROTEM assessment. In several studies, hypercoagulable conditions were identified using ROTEM in disease states with an increased risk of thromboembolic events [15, 16]. Moreover, viscoelastic systems, such as ROTEM and thromboelastography, were successfully established to detect hypo- or hyperfibrinolysis in patients with traumatic injury or severe septic shock [17, 18].

Panigada et al. used thromboelastography in 20 patients with COVID-19 in addition to plasmatic tests of coagulation [19]. Similar to our study, they also found increased levels of fibrinogen and factor VIII, and almost normal routine coagulation tests. Thromboelastography data showed elevated clot firmness as reflected by maximal amplitude and reduced fibrinolysis measured as reduced clot lysis at 30 min (Lys 30), consistent with our observations. Spiezia and colleagues and Pavoni and co-workers also recently showed severe hypercoagulopathy in critically-ill COVID-19 patients using ROTEM [20, 21]. They found a significantly higher maximal clot firmness in INTEM, EXTEM and FIBTEM, and shorter INTEM clot formation time in comparison with a healthy control group. However, they observed no differences between COVID-19 patients with and without thrombosis [20]. In a cohort of 19 patients, Ibañez et al. noted markedly reduced fibrinolysis in COVID-19 patients; however, no distinction with respect to the presence of thromboembolic events was made [23].

While our findings confirm these results, we noted not only a markedly reduced fibrinolysis in the whole cohort but a significantly reduced ML in the group with thromboembolic complications. The clot lysis parameter ML provides information on the fibrinolytic activity, with low values providing evidence for hypofibrinolysis. In the current study, we found the ML in both EXTEM and INTEM to be markedly below normal values. Furthermore, the ML under both conditions was even lower in the group with thromboembolic complications. Therefore, we conclude that a severely impaired fibrinolysis plays an important role in the hypercoagulable state and thromboembolic risk in COVID-19 patients [23].

It is, however, somewhat surprising that highly elevated levels of D-dimers were found in a state of hypofibrinolysis. As a hypothesis, it has been suggested that intra-alveolar fibrin deposition accounts for local activation of fibrinolysis in ARDS.

The mechanisms leading to hypofibrinolysis in COVID-19 remain to be defined. Complex interactions between inflammation and the coagulation and fibrinolytic system have been examined and controversially discussed for decades [24–26]. One potential mechanism may be the production of alpha defense in neutrophils, which are known to promote fibrin polymerization and block fibrinolysis in vitro [27].

In our cohort, we found markedly elevated markers of inflammation, including interleukin-6, CRP and ferritin; however, only the maximum CRP level differed significantly between patients with and without thromboembolic complications. We could not detect significant differences among additional individual analytes (i.e., tPA or PAI concentrations) between both groups; however, we did not evaluate the effect of the complement or bradykinin system, which are both known to play crucial roles in connecting the inflammatory response and fibrinolytic activity. Future clinical trials should also focus on the role of thrombin-activatable fibrinolysis inhibitor (TAFI), plasmin-alpha-2-antiplasmin (PAP) complexes and antiplasmin, which would give valuable insights into the mechanisms of COVID-19-induced hypofibrinolysis. Furthermore, endothelial dysfunction is likely involved but was not assessed.

ROC analyses provided an AUC for ML in EXTEM of 0.8. As such, it might be a candidate as prediction marker of future thromboembolic complications. Zhou et al. reported D-dimers to be one of the most sensitive and specific factors predicting mortality in a large cohort of COVID-19-patients in China [14]. Cui et al. found a good sensitivity and specificity using a cutoff of 1.5 ng/ml for predicting thrombotic events in COVID-19 patients [8]. D-dimers were also markedly elevated in our cohort and were found to be significantly higher in the subgroup with thromboembolic events. ROC analysis for D-dimers revealed an AUC of 0.78. The combination of the maximum D-dimer and ML in EXTEM (D-dimer—ML) improved the AUC to 0.92, with a cutoff of 3.7 for a sensitivity of 94% and specificity > 90%. The predictive value of this D-dimer–ML parameter, however, requires validation in a second cohort.

In addition to providing insights in the mechanism of thrombus formation, our results may underline the possible therapeutic option of specific fibrinolytic therapy for ARDS caused by COVID-19. Administration of recombinant t-PA has already been suggested as a potential treatment and has shown promising results in a previous study independent of COVID-19 [28]. Currently, a phase IIa trial is underway to examine the effect of thrombolytics in COVID-19 patients with hypoxemic lung injury (ClinicalTrials.gov, NCT 04357730) [29].

There are several limitations to our study. First, ROTEM measurements were performed when patients were transferred to our ICUs after different treatment periods in other hospitals. Thus, the ROTEM results reflect different stages of the disease. Also, many, but not all patients, were previously treated with heparin when thromboelastometry measurements were performed. Second, the study is monocentric, performed in a tertiary care center, and the generalizability to other settings and patients with a less severe course and earlier stages of the disease needs to be tested. Third, our prediction models based on associations between poor clot lysis, D-dimers and the presence of thromboembolic events are hypotheses and require validation in independent patient cohorts and prospective observational studies. Fourth, thromboembolic events may have been underdiagnosed, as only ultrasound was routinely performed, while CT scans to exclude pulmonary embolism were only performed in some patients. Fifth, our results are descriptive in nature and do not provide explanatory models for the observed hypofibrinolysis. Future studies should focus on the examination of possible mechanisms.

Sixth, 25% of patients of our cohort received ECMO therapy, which may itself have had a thrombogenic effect and in part may have contributed to the high rates of thrombosis. However, the current literature points into the direction that in some cases ECMO rather leads to hyperfibrinolysis [30]. An ECMO-side effect as an explanation for a systematic hypofibrinolysis as observed in our cohort thus appears rather unlikely. Seventh, even though the statistical analysis showed robust values for our analysis, it may be difficult to guide clinical decision based on these values, as the difference in maximum lysis is 2%.

In summary, we found substantially reduced fibrinolysis in COVID-19 patients, which was more pronounced in patients with thromboembolic events. Clot ML time, as assessed by ROTEM as a single parameter, or in combination with D-dimers may prove valuable for thromboembolic risk stratification in COVID-19 patients and aid in decision-making regarding anticoagulation strategies.

## Conclusions

ROTEM revealed severe hypofibrinolysis in COVID-19 patients. Maximum lysis, especially following stimulation of the extrinsic coagulation system, was inversely associated with an enhanced risk of thromboembolic complications. The combination of maximum lysis with D-dimer concentrations revealed high sensitivity and specificity of thromboembolic risk prediction. Hence, ROTEM may help to identify patients benefiting from therapeutic anticoagulation.

## Data Availability

The data that support the findings of this study are available from the corresponding author upon reasonable request.

## Abbreviations

ROTEM: Rotational thromboelastometry
COVID-19: Coronavirus 2019
SARS-CoV-2: Severe acute respiratory syndrome coronavirus 2
ARDS: Acute respiratory distress syndrome
ICU: Intensive care units
MCF: Maximum clot firmness
ML: Maximum lysis
PT: Prothrombin time
INR: International normalized ratio
aPTT: Activated partial thromboplastin time
t-PA: Tissue-type plasminogen activator
PAI-1: Plasminogen activator inhibitor-1
IQR: Interquartile range
ROC: Receiver operating characteristic
AUC: Area under the curve
CI: Confidence intervals
PCT: Procalcitonin
CRP: C-reactive protein
